# Gold Metal Recovery from Electronic Waste through Laser Generation of Micro and Nanoparticles

**DOI:** 10.1021/acsomega.5c02781

**Published:** 2025-06-09

**Authors:** Abhishek Trivedi, Mahantesh Khetri, Atchutananda Surampudi, Mool C. Gupta

**Affiliations:** Charles L. Brown Department of Electrical and Computer Engineering, University of Virginia, 351 McCormick Road, Charlottesville, Virginia 22904, United States

## Abstract

Electronic waste (E-waste) is the fastest-growing waste stream globally, reaching 74.7 tonnes by 2030, containing significant amounts of valuable metals such as gold, silver, platinum, and copper. Mechanical, hydrometallurgical, pyrometallurgical, electrochemical, and biotechnological methods for recovering these metals from E-waste are often inefficient, costly, and environmentally harmful. This study presents the first demonstrations of laser ablation in recovering gold in the form of micro and higher-valued nanoparticles from E-waste. The ablation threshold is identified using modeling performed using the two-temperature model (TTM). Printed Circuit Boards (PCBs) with gold-plated electrodes were used as the target material. The laser ablation process was conducted using a picosecond UV-355 nm laser at maximum average laser power (18 W). The analysis, using UV–visible spectroscopy, shows the surface plasmon resonance peak at 523 nm for gold nanoparticles­(Au NPs), and SEM–EDX mapping confirmed the successful creation of high-purity (90 wt %) Au NPs with an average size of 100 nm. Laser-Induced Breakdown Spectroscopy (LIBS) was used to monitor the elemental composition of the E-waste sample during the ablation process to demonstrate real-time processing monitoring. The ability to recover gold in nanoparticle form further enhances the economic viability of this technique giving a wide range of applications for gold nanoparticles in various fields. The findings underscore the potential of laser ablation as a sustainable solution for E-waste recycling, addressing critical global challenges related to the recovery of valuable materials.

## Graphical abstract

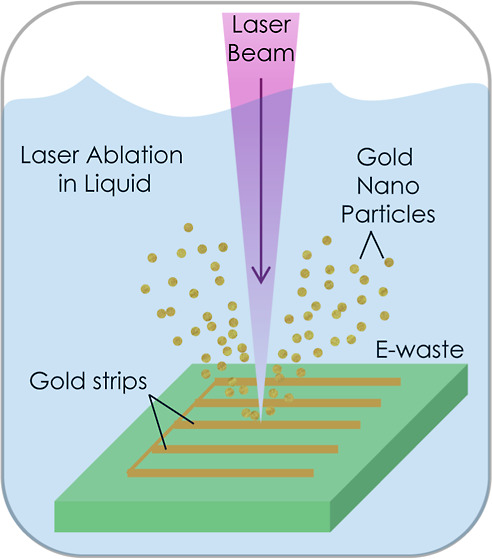

## 1 Introduction

E-waste is the world’s fastest-growing waste stream, with an annual growth rate of 3–5%, and by 2030, it will reach 74.7 tonnes.^1,2^ E-waste contains precious metals like gold, silver, platinum, and copper, which have high electrical conductivity and corrosion resistance and have been used for various electronic devices.^3^ The total value of materials in global E-waste in 2022 is estimated at 91 billion dollars, of which gold has a value of 15 billion dollars,^4^ and the volume of gold in global E-waste is approximately 0.1% of the other materials.^5^ The gold concentration in E-waste is ∼250 g/ton and the gold concentration in other waste associated with E-waste lies in the range of ∼1–10 g/ton.^6^ Unfortunately, these precious metals are often lost when E-waste is discarded without recycling. Gold is highly valuable, making its recovery from E-waste financially attractive. Figure 1a shows the projected waste generation worldwide from 2019 to 2030, and Figure 1b shows various applications of gold nanoparticles. Recycling gold from E-waste reduces the need for costly and environmentally damaging gold mining, which involves extensive excavation, chemical processes, and substantial water and energy use, leading to habitat destruction, soil erosion, pollution, and carbon emissions.^7^ PCBs, connectors, hard drives, pins, integrated circuits, and memory modules are some of the electronic devices that contain thin gold films deposited by an electroplating process.^8^ The gold content in electronic devices varies significantly depending on application requirements. Thin gold plating (0.1–0.5 μm) is suitable for static contacts, while moderate thicknesses (1.25–2.5 μm) enhance durability and conductivity in dynamic connectors. Military and aerospace applications often require thicker gold layers (12.5–25 μm). All these gold types can be utilized for recycling purposes.^9^

**Figure 1:**
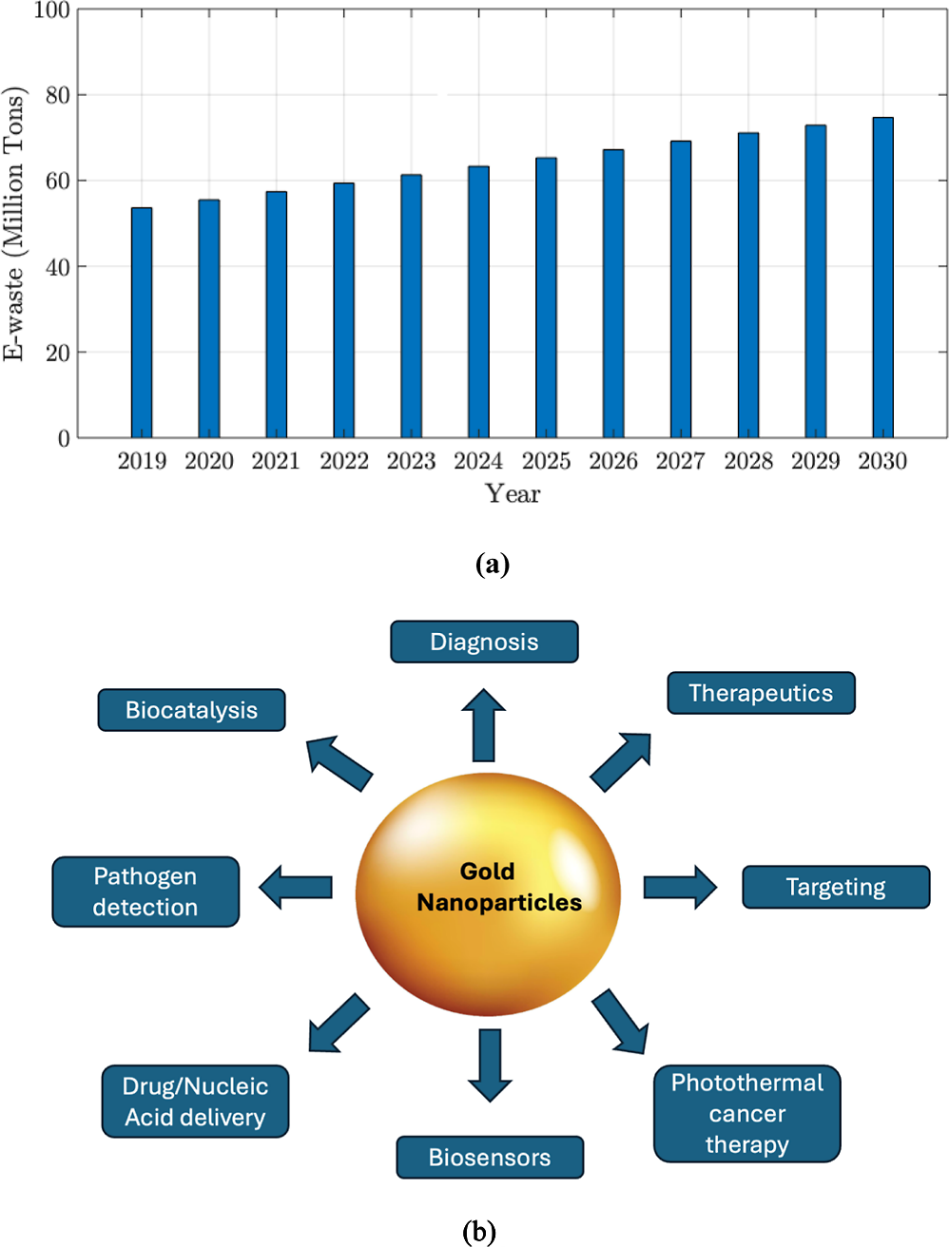
(a) Estimated global E-waste generation by 2030. Data obtained from source^10^ https://www.statista.com/statistics/517449/large-household-appliances-ewaste-uk/ (b) Various applications of Au nanoparticles. Reprinted with permission from,^11^ Copyright 2022, MDPI Chemistry.

There are several existing methods for recovering gold from E-waste, including mechanical, hydrometallurgical, pyrometallurgical, electrochemical, and biotechnological.^12^ In the mechanical method, the PCBs go through mechanical treatment like crushing, shredding, and grinding using techniques like crushers, sieves, wind separators, magnetic separators, eddy current separators, conveyors, and dust collectors, which break down the E-waste into smaller particles and it separates gold from other components. Approximately 0.1 g of gold can be recovered per 1 kg of PCBs and about 1970 kWh of electricity consumption^7^ in the first step, and in the next step, the separated gold undergoes further refinement using inverse aqua regia (3:1 HCl: HNO_3_ mixture) and 15.5 L of aqua regia is used per kg of recovered gold. The process results in a yield of 98 wt % gold powder.^13^

The hydrometallurgical process for E-waste recycling involves multiple leaching steps to extract valuable metals. Initially, the E-waste is pulverized to reduce particle size. Subsequent leaching stages utilize various chemical solutions, including sulfuric acid with hydrogen peroxide, thiourea with ferric iron, and a chloride-based mixture.^14^ Each leaching step is followed by solid–liquid separation. The process concludes with precipitation stages using sodium borohydride as a reducing agent to recover gold and silver from the leach solutions. This method, while effective for metal recovery, requires about 2868 kW h of electrical energy to recover one kilogram of gold.^15^ Similarly, Pyrometallurgical, electrochemical, and biotechnological methods for gold recovery from E-waste are multistep processes that employ various chemical reactions under different temperature conditions. These methods differ significantly in their energy consumption, efficiency, and environmental impact. For instance, pyrometallurgy involves high-temperature operations (typically 1300–1800 °C) that consume substantial amounts of electricity but offer rapid processing.^16^ Electrochemical methods, while often more energy-efficient, may require longer processing times and specific electrolyte compositions.^17^ Biotechnological approaches, such as bioleaching, are generally less energy-intensive but can take days or weeks to complete and require carefully controlled environmental conditions for optimal microbial activity.^18^ Each method presents unique challenges in terms of worker safety and environmental hazards. Pyrometallurgy poses risks related to high temperatures and potential toxic emissions.^19^ Electrochemical processes often involve corrosive electrolytes and generate potentially harmful byproducts.^20^ Biotechnological methods, while generally safer, may still require handling acidic solutions or potentially pathogenic microorganisms.^21^ This research paper presents an alternative technique of laser ablation for recycling gold from E-waste.

Laser processing is a technique that offers a rapid, chemical-free approach^22^ to recovering gold in valuable nanoparticle form. This method uses powerful laser pulses to remove gold from E-waste. When the laser hits the gold-plated electrodes of PCB (E-waste), it vaporizes the gold, and the condensation of gold vapor in the air produces microparticles, in water, the gold vapor expands, forming a cavitation bubble. Upon the collapse of this bubble,^23^ the gold particles cool down and form Au NPs. Using this method, the size and shape of both nanoparticles and microparticles can be tuned by adjusting laser parameters such as pulse energy, repetition rate, and ablation time, as well as the properties of the surrounding medium.

In this manuscript, we present results of a new approach for E-waste recycling using a laser ablation process in liquid to obtain higher valued gold nanoparticles. First, a laser–gold interaction was modeled using a two-temperature model to identify the optimal ablation threshold for UV–355 nm picosecond pulses. Laser ablation experiments on gold–plated PCB samples under 1.5 mm water immersion were carried out. The resulting micro- and nanoparticles were collected and analyzed via UV–Vis spectroscopy for determination of nanoparticle size, SEM–EDX mapping for morphology, size, purity, and LIBS for real-time layer-by-layer process monitoring. Additionally, a cost analysis of the laser process for E-waste recovery was carried out, and the results indicate a highly economical and environmentally sustainable process.

## 2 Modeling

Gold recovery from electronic waste via laser ablation relies on precise control of laser–material interaction parameters. The process is dictated by factors such as laser wavelength, pulse duration, laser power, spot size, and the material’s optical and thermal properties. These parameters govern how efficiently gold absorbs the laser energy, leading to localized heating, melting, and phase explosion, which drive the ablation process.^24^ By carefully adjusting these settings, gold can be selectively removed while minimizing damage to surrounding materials.

### 2.1 Influence of Laser Wavelength

In this study, a 355 nm ultraviolet (UV) picosecond laser with a 15 ps pulse duration was used to investigate the ablation behavior of gold. The ability of gold to absorb laser energy is strongly influenced by its reflectance, which varies with wavelength. The reflection coefficient at normal incidence is given by

1 $$R=\frac{{\left(n-1\right)}^{2}+k^{2}}{{\left(n+1\right)}^{2}+k^{2}}$$

where *n* and *k* are the real and imaginary parts of the complex refractive index of gold. A plot of reflectance vs the wavelength was simulated, as shown in Figure 2, with the support of the Filmetrics KLA software.^25^

**Figure 2:**
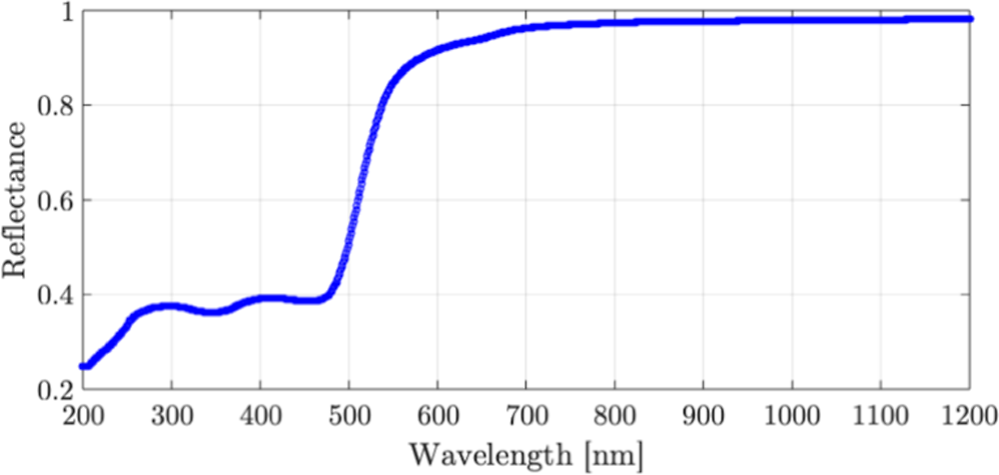
Reflectance vs wavelength for a gold film at normal incidence.

At infrared wavelengths, gold exhibits a high reflectance (∼97%), significantly reducing energy absorption. In contrast, in the UV wavelengths, for example, at 355 nm, reflectance drops to approximately 36%, allowing for greater absorption and more efficient ablation. This stark contrast demonstrates why in this manuscript the UV lasers have been used, as they deliver energy with minimal reflection losses.

The optical penetration depth δ, determining how deep laser energy is absorbed, is given by

2 $$\delta =\frac{\lambda }{4\pi k}$$

where λ is the laser wavelength and *k* is the extinction coefficient. For gold, at 355 nm laser wavelength, the extinction coefficient is 1.75. So, the depth is calculated as 16 nm which is extremely shallow, ensuring that energy remains concentrated at the surface. This characteristic is particularly useful in removing thin gold coatings from substrates while preventing excessive thermal damage.

### 2.2 Picosecond vs Nanosecond Pulses

Ablation behavior varies considerably between nanosecond and picosecond laser pulses. The spatial extent of thermal effects is quantified by the thermal diffusion length, given by

3 $$L_{T}=2\sqrt{{\alpha t}_{p}}$$

where α is the heat diffusion coefficient of gold^26^ (α = 1.27 × 10^–4^ m^2^/s). A nanosecond laser with a 10 ns pulse width, produces a thermal diffusion length of 2.25 μm, allowing deeper heat penetration that can lead to unwanted substrate modifications, which can further lead to large area melting and resolidification rather than direct vaporization. This results in inefficient ablation and increased thermal damage to the underlying substrate. In contrast, a picosecond laser, with a 15 ps pulse duration (used in the experiment), has a diffusion length of 87 nm, enabling highly localized ablation without affecting the underlying material, with minimal heat diffusion. The resulting material removal is clean and precise, making picosecond pulses ideal for high-purity nanoparticle generation.

The rise in temperature of the surface of the gold film using a picosecond laser can be modeled using the two-temperature model (TTM),^27^ which helps quantify the ablation threshold of the pulse energy per unit area for the gold film. The TTM model describes the increase in the metal lattice temperature due to the electron–phonon coupling, following the electron energy absorption. For the TTM model, we calculate the electron temperature. In this regard, for a specific heat capacity *C*_e_ of electron absorption, we first calculate the rise in electron energy per unit volume *Q*_e_, which is expressed as

4 $$Q_{e}=\int_{T_{o}}^{T_{e}}C_{e}\;dT=\int_{T_{o}}^{T_{e}}\gamma T\;dT=\frac{\gamma }{2}\left(T_{e}^{2}-T_{o}^{2}\right)$$

where it is assumed that the specific heat capacity *C*_e_ is temperature dependent with γ being the electron heat capacity coefficient (in J/m^3^ K^2^), and *T*_e_ and *T*_o_ respectively being the final electron temperature and the initial ambient temperature. The rise in electron energy^28^ per unit volume *Q*_e_ can also be expressed as

$$Q_{e}=\left(1-R\right)\times \frac{P_{peak}\tau }{A\delta }$$ 5

where *R* is the reflectivity, *P*_peak_ is the peak power of the laser pulse, τ is the electron absorption time, and *A* is the diffraction-limited area of spot size impinged by the laser, with δ being the absorption depth. Combining eqs 4 and 5, we have

$$T_{e}=\sqrt{T_{o}^{2}+\frac{2\left(1-R\right)P_{peak}\tau }{A\delta }}$$ 6

Following the electron temperature rise to *T*_e_, as expressed in eq 6, the rise in the lattice temperature can then be expressed as

7 $$C_{e}\frac{{\partial T}_{e}}{\partial t}=-G\times \left(T_{e}-T_{l}\right)+\left(1-R\right)\frac{F_{laser}}{\delta },,C_{l}\frac{{\partial T}_{l}}{\partial t}=G\times \left(T_{e}-T_{l}\right)$$

where *C*_l_ and *T*_l_ are respectively the specific heat capacity and the temperature of the gold lattice, G is the electron–phonon coupling coefficient, and *F*_laser_ is the laser energy per unit area (fluence) on the gold surface of the impinged by the laser. The temperature of the lattice T_l_ reaches an equilibrium beyond a time τ_e_ expressed as *C*_e_/*G*, after which the spatial diffusion of heat starts according to the heat diffusion expression

8 $$\frac{\partial T}{\partial t}=\alpha \nabla^{2}T$$

where α is the heat diffusion coefficient of gold (in m^2^/s), as also mentioned for the eq 3. These equations were simulated in Matlab, with eq 8 performed using Laplacian central differences. Physical constants were assumed from ref 26. The temperature vs time simulated plots for different peak power % of the laser are shown in Figure 3a,b, respectively over picoseconds and nanoseconds time scales. The limitations of the modeling are as follows. The temporal shape of the pulse is assumed a binary square pulse, which simplifies the assumptions involved in modeling. The temperature dependence of the specific heat constants is not considered for brevity in calculations. In eq 7, for the two-temperature modeling, the diffusion in the *z*-direction has not been considered to allow for simplicity in the computation.

**Figure 3:**
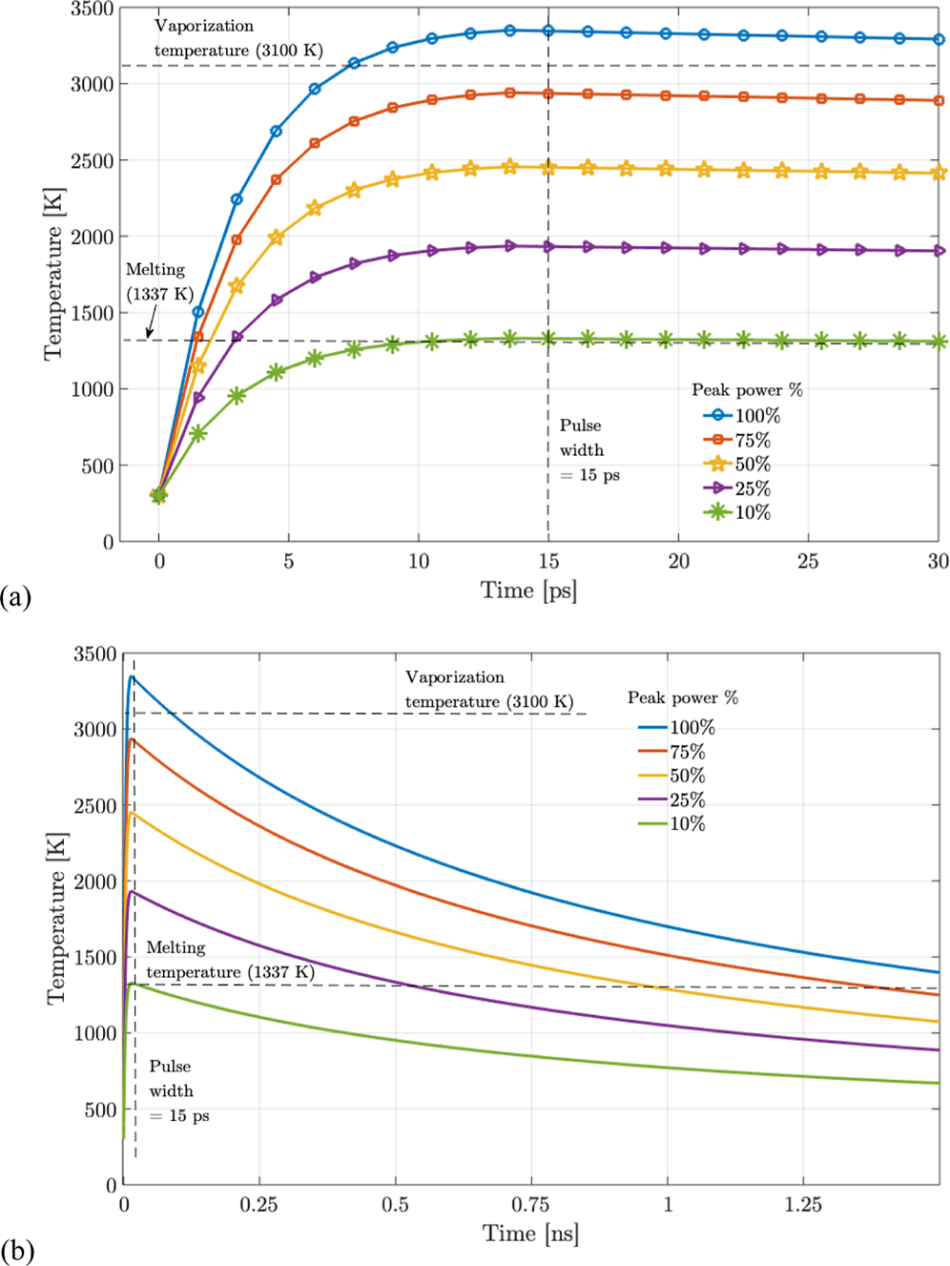
Temperature vs Time simulation in Matlab for (a) ∼ps, (b) ∼ns. A peak power of 100% equals 3.3 MW of the picosecond pulse.

From Figure 3a, it can be observed that below 10% peak power, it results in heating with almost reaching the melting temperature. At 25–50% peak power, the melting occurs but does not reach the vaporization temperature for nanoparticle formation. Beyond 75% peak power, temperatures >3000 K are achieved allowing nanoparticle formation. As a note, the simulation parameters for the laser are peak power of 3.3 MW, pulse duration of 15 ps, a Gaussian spatial profile of the beam, focused by an objective with a numerical aperture of 0.5, which is the same as the experimental setup parameters in the manuscript. In Figure 3a, it can be further observed that the temperature rise reaches an equilibrium in the order of a ∼few ps, and beyond the pulse duration of 15 ps the temperature drops due to spatial diffusion (as expressed in eq 8), as shown in Figure 3b.

These simulations help note the threshold % of peak power needed to form nanoparticles. Based on these simulations, so for the experiments, a peak power of >75% is used to obtain the results in the manuscript.

### 2.3 Optimization of Processing Parameters

Efficient gold ablation depends on optimizing laser fluence, repetition rate, and scanning speed. Higher fluences ensure complete vaporization but must be carefully controlled to avoid excessive heating. The repetition rate dictates how frequently laser pulses interact with the material, influencing overall removal rates. The scanning speed must be balanced to allow uniform ablation while preventing overexposure to thermal energy.^29–32^ To enhance ablation efficiency, a cooling medium such as water is often employed. Water immersion prevents overheating, facilitates nanoparticle collection, and promotes rapid cooling of vaporized gold, yielding high-purity nanoparticles.^33^ By fine-tuning laser parameters, gold ablation from E-waste can be performed with high precision, maximizing material recovery while minimizing collateral damage. This selective removal approach enables sustainable and efficient extraction of valuable gold from discarded electronics, contributing to improved recycling processes.

## 3 Materials and Equipment

For the laser ablation experiment, memory modules were used, specifically RAM PCBs with gold-plated electrodes shown in Figure 4. Each RAM device sample featured double-sided gold plating, with each side comprising 121 gold-plated electrodes. Each electrode measured 2.3 mm in length and 1 mm in width, with an average thickness of 43 μm. The dimensions of the samples were measured using a Hirox Digital Light Microscope (as shown in Figure 4b), and the electrode thickness was measured by performing a cross-section analysis of the sample using the optical microscope as shown in Figure 4c with a magnification of 5×.

**Figure 4:**
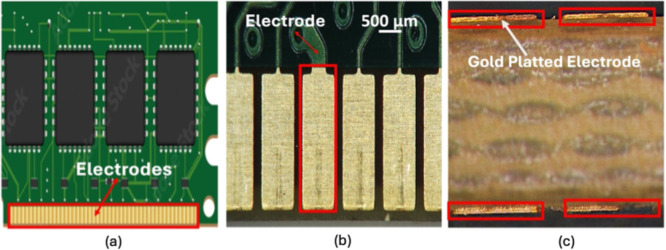
(a) Photograph of the PCB with the gold-plated electrodes (in red rectangle box), (b) magnified image of gold-plated electrodes (in red rectangle box). (c) Cross-section image of PCB sample (in red rectangle box). Magnification = 5×.

The laser ablation setup schematic is shown in Figure 5. The laser ablation was conducted using the Spectra-Physics IceFyre UV30 ps laser, which operated at a wavelength of 355 nm with a pulse width of 15 ps and a pulse energy of 50 μJ. The laser beam exhibited a Gaussian beam profile with a full width at a half-maximum (fwhm) size of 60 μm when focused on the sample surface. For the characterization of the light absorption properties of the solution containing nanoparticles, a Cary 5E UV–vis-NIR spectrometer was employed. The morphology and elemental composition of the nanoparticle samples were analyzed using a FEI Quanta 650 field emission Scanning Electron Microscopy (SEM) with Energy Dispersive X-ray (EDX) spectroscopy attachment. Nanoparticle samples for SEM–EDX mapping were prepared by depositing a drop of the nanoparticle-dispersed solution onto a silicon wafer.

**Figure 5:**
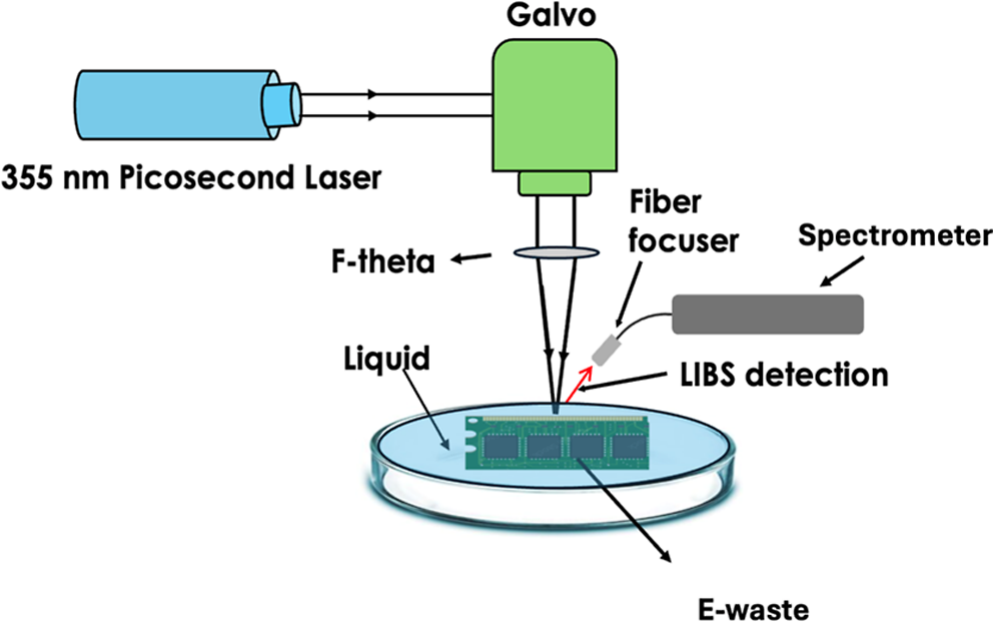
Schematics of the experimental setup.

## 4 Results and Discussion

### 4.1 Characterization of Au-Plated Electrode after Laser Ablation

We performed laser ablation on electronic waste samples to analyze the material layers present on the electrodes. As shown in Figure 6, the laser was used to remove the gold layer, revealing the underlying nickel layer, which can be seen in Figure 6a,b. By further applying laser ablation to the nickel, we were able to reach the bottom layer of copper, as shown in Figure 6c. This process allowed us to confirm that the electrode consists of gold as the top layer, nickel as the middle layer, and copper as the bottom layer. The gold thickness^34^ is 0.5–1.27 μm, the nickel thickness^24,35^ ranges from 3 to 6 μm, and the copper thickness^36^ is between 20 and 35 μm.

**Figure 6:**
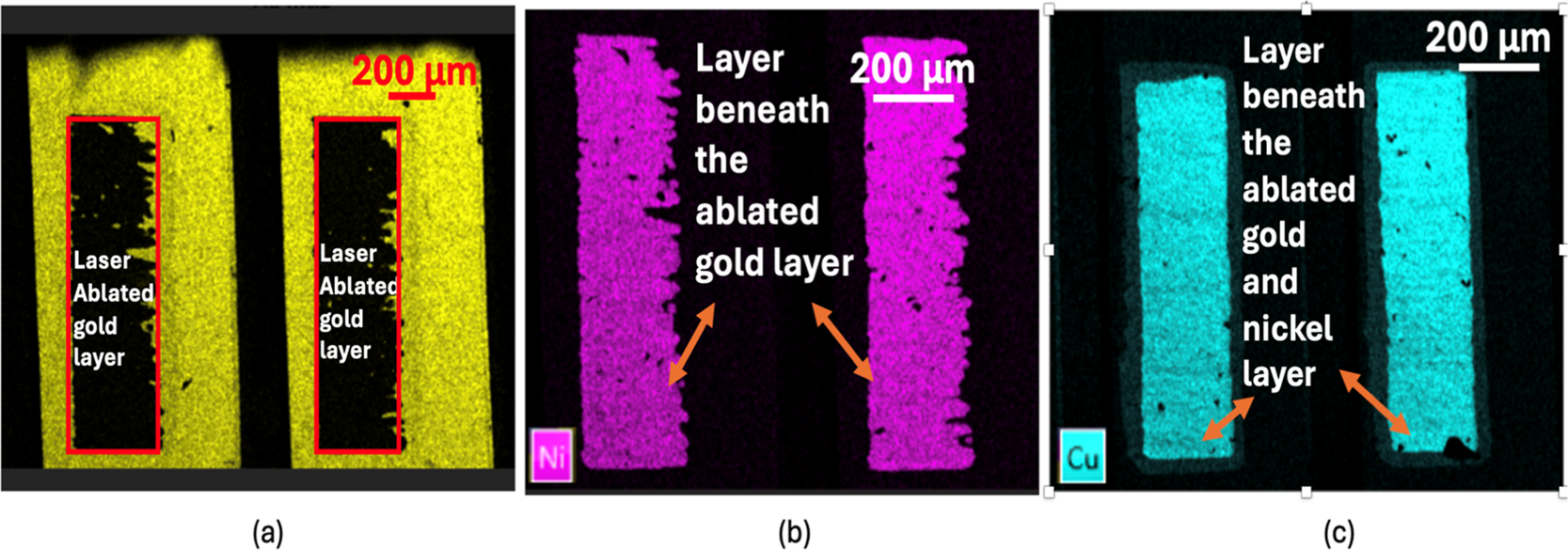
(a) EDX mapping for gold illustrating the removal of the gold layer. (b) EDX mapping for nickel reveals the underlying nickel layer after removing gold. (c) EDX mapping for copper reveals the underlying copper layer after removing gold and nickel.

### 4.2 Characterization of Au Nanoparticles Generated by Laser Ablation Process

In this study, gold nanoparticles (Au NPs) were synthesized using laser ablation in a liquid medium with the 15 ps UV-355 nm picosecond laser. During the experiment, the water layer above the sample was maintained at a thickness of 1.5 mm, and the laser scanning speed was set to 500 mm/s. UV–vis spectroscopy was used to analyze the resulting colloidal solutions, with absorbance plotted against wavelength, as shown in Figure 7. The spectrum consistently showed a characteristic surface plasmon resonance (SPR) peak at 523 nm, and the full width at half-maximum (fwhm) is measured as 45 nm, confirming successful Au NP formation.^37^

**Figure 7:**
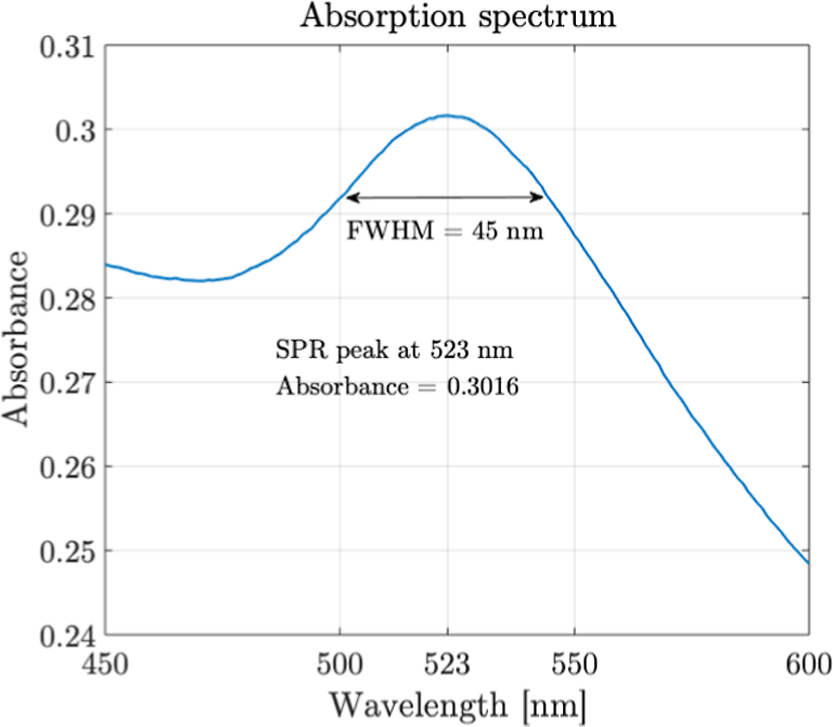
Absorption spectrum of gold nanoparticles. This was obtained over 5 laser scanning passes, using a 100% laser average power of 18 W and a scanning speed of 500 mm/s.

The nanoparticles in colloidal solution were characterized using SEM–EDX mapping, as shown in Figure 8. From Figure 8a, the mapping depicts spherical nanostructures of Au particles with a size ranging from 50 to 200 nm in diameter with a purity of around 90 wt % (as shown in Figure 8c), and with an average size corresponding to 100 nm, which supports the 523 nm SPR peak. Oxygen was detected as the major impurity in the gold nanoparticles shown in Figure 8b. The presence of oxygen could be assigned to the hydrocarbons in the PCB.^38^ These impurities can arise from atmospheric exposure during synthesis, residual contaminants on the PCB, or laser ablation of areas outside the gold-platted electrode due to laser misalignment. To minimize these impurities, several strategies can be employed: conducting the synthesis in an inert atmosphere to prevent oxidation, thoroughly cleaning the E-waste before processing, and using centrifugation after synthesis. Centrifugation involves rapid spinning to create a force that causes heavier particles to settle at the bottom of the container, facilitating cleaner collection of the nanoparticles.

**Figure 8:**
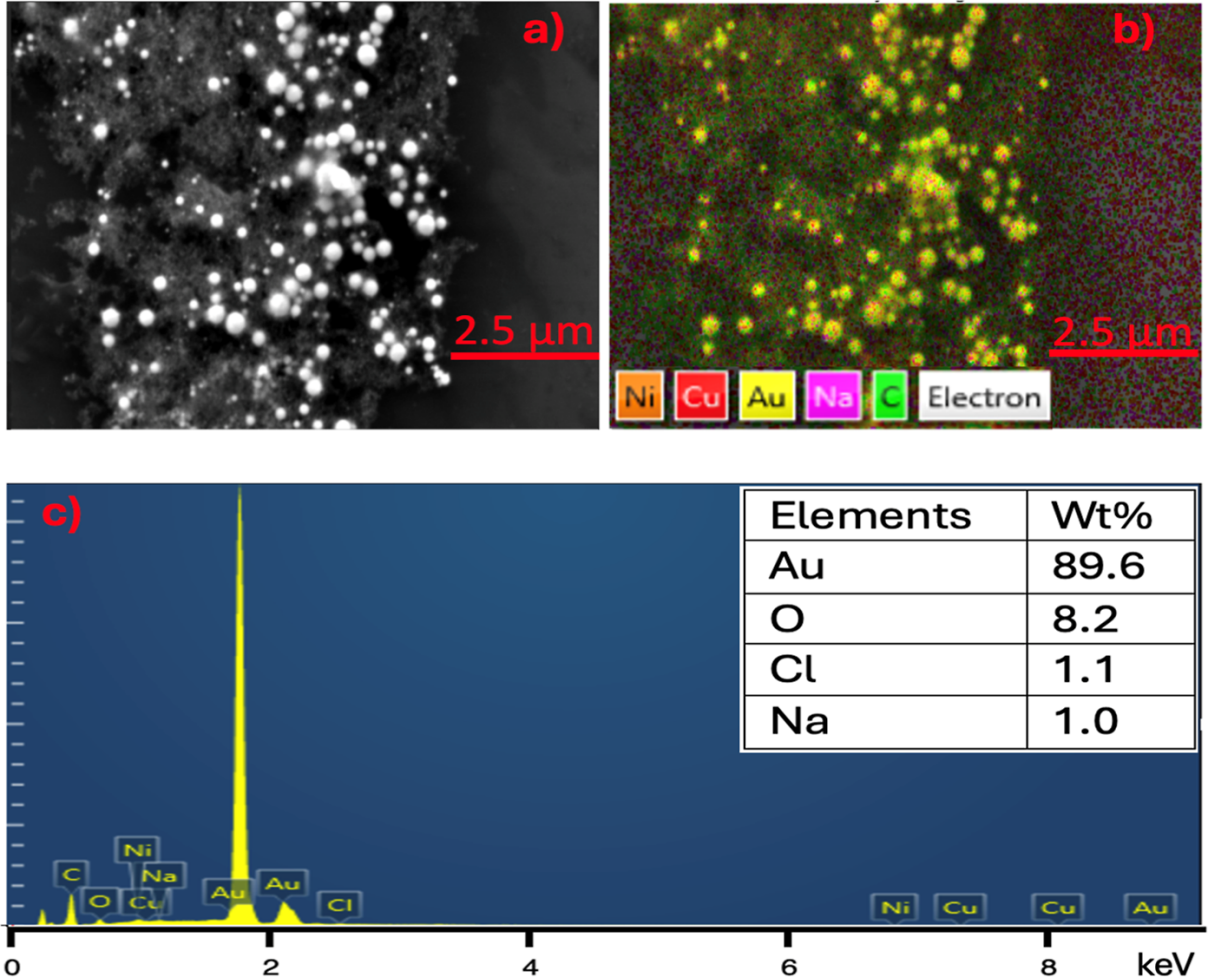
(a) SEM image of laser-ablated Au nanoparticles. (b) EDX mapping showing the distribution of Au nanoparticles. (c) Elemental composition analysis confirming the presence of Au.

### 4.3 Process Monitoring with Laser-Induced Breakdown Spectroscopy

The process of using a laser to ablate and generate nanoparticles allows LIBS to be used as a method to observe the elemental composition of the target sample simultaneously during the ablation process. Figure 9 shows the measured spectra over different passes and different powers −1, 10, and 20 and 50% and 100% average power (18 W). In the case of 1 pass (50% power) in Figure 9a, the Au gold peak^29^ at 312 nm is observable, which confirms the ablation of gold strips without any contamination. As the number of passes increased, at 10 passes, it could be observed in Figure 9b, that LIBS peaks in the range of 290 nm could be observed along with the LIBS peak of Au. This additional peak at 290 nm corresponds to the Ni II transitions of nickel compared to its strong peaks in the 300 nm and above spectral range, occurring due to the ionization of singly ionized Ni during plasma generation. This is expected due to the layer-by-layer composition of the E-waste sample (RAM modules) used in the experiment (in the order of Au, Ni, and Cu, starting from the top surface). In this regard, allowing more passes, such as 20 passes as shown in Figure 9c, the LIBS peak of Au at 312 nm significantly reduces, along with the emergence of Ni I LIBS peaks beyond the 300 nm wavelength range, indicating the presence of Ni and Cu on the sample. The experiment is repeated to observe this phenomenon at a higher laser power (100%, 18 W). In this experiment, in the first pass, as shown in Figure 9d, the Ni LIBS peak at 290 nm comes up along with the peak of Au at 312 nm, which is expected because an increase in the laser power ablates the material deeper in a single pass itself. When more passes are allowed, for example, in 10 passes, as shown in Figure 9e, a bulk of the top layer material is removed, and there is an emergence of other peaks beyond the 300 nm wavelength range with the disappearance of Au peak (similar to the case in Figure 9c with 50% power with more passes). These experiments summarize that the LIBS method can be applied to control the purity of laser-ablated Au micro and nanoparticles.

**Figure 9:**
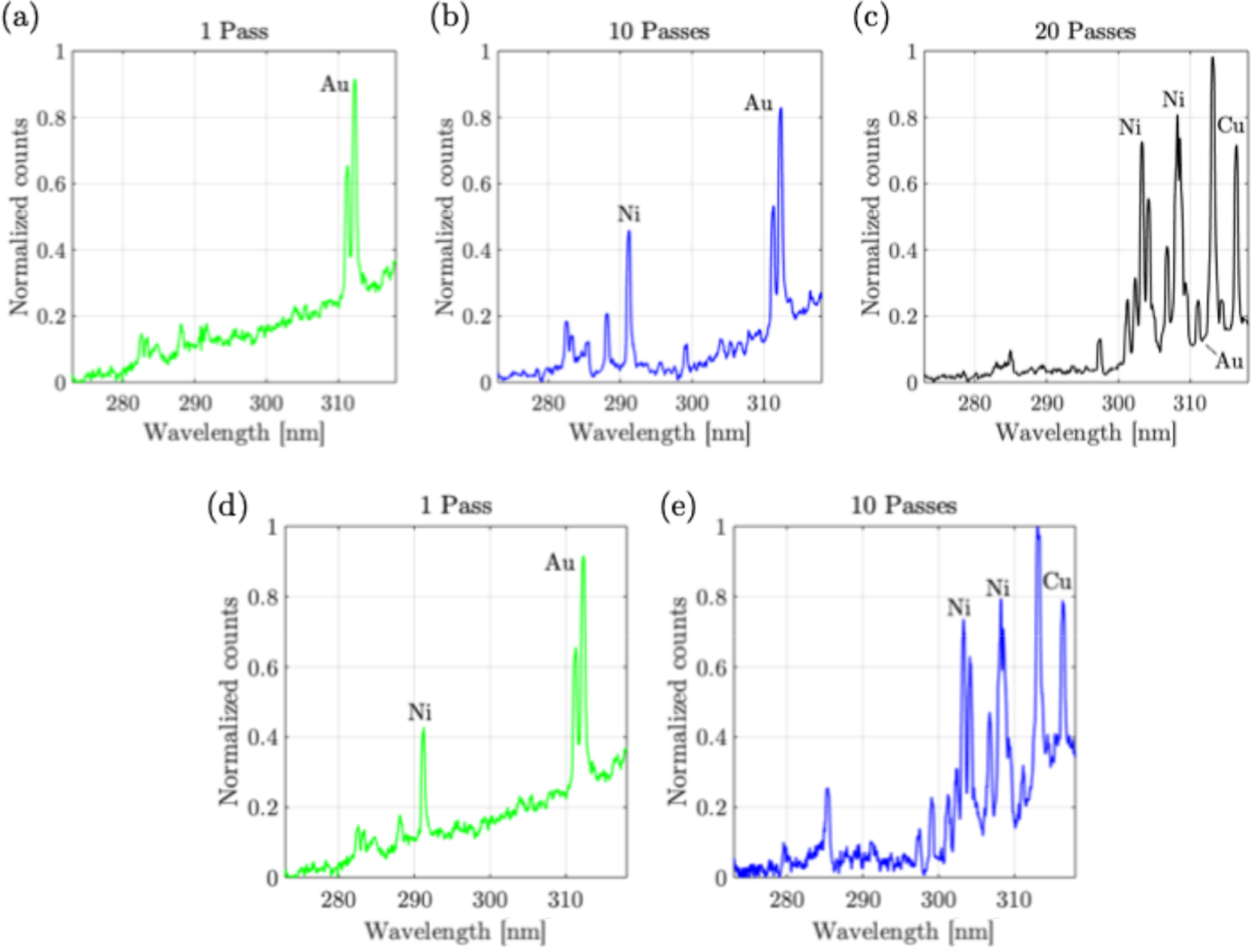
Process monitoring with LIBS at different laser scanning passes—(with 50% power, 9 W) (a) 1 pass, (b) 10 passes, (c) 20 passes; (with 100% power, 18 W) (d) 1 pass, (e) 10 passes.

### 4.4 Cost Estimation for the Gold Recovery from E-Waste

A preliminary cost analysis was performed for gold recovery from RAM PCBs using a laser ablation process. Each PCB contains 242 gold-plated strips, measuring 2.3 mm in length and 1 mm in width, with a normal 0.5–0.8 μm gold layer thickness.^39^ The total gold content per PCB was calculated to be 5.3–8.6 mg. The recovery process takes approximately 15 s per PCB, utilizing a laser scanning speed of 500 mm/s and 50 scans per gold-platted electrode. Assuming 90% purity, the gold yield per PCB is estimated at 4.84–7.74 mg.

The laser process consumes an average of 300 W of electrical power, including all electronic equipment and the laser, resulting in an operational cost of 0.04 USD per hour based on a commercial electricity rate of 0.13 USD per kWh. Considering the initial investment of 50,000 USD for the laser system and its anticipated lifespan of 10 years (or over 100,000 h of operation), the total operational cost is estimated to be 0.50 USD per hour. This translates to a gold recovery cost of approximately 0.18 USD per gram. In contrast, the current market price of gold ranges from 70 to 80 USD per gram, while gold nanoparticles command a premium of 100 to 150 USD per gram. This analysis demonstrates the economic viability of the laser ablation process for gold recovery from E-waste, highlighting the significant value of the recovered material.

## 5 Conclusions

In summary, this study has presented the first demonstration of the recovery of gold from electronic waste using the process of laser ablation in liquid. The experiment was first modeled in Matlab using the two-temperature model (TTM) to identify the threshold of the % of peak power needed to perform the laser ablation and generation of nanoparticles. The technique was thereafter successfully demonstrated as a cost-efficient and environmentally friendly technique that leverages the capabilities of UV-355 nm lasers for the ablation of gold-plated electrodes from PCBs, which can be further extended to other metallic waste as well. The analysis, involving UV–visible spectroscopy and SEM–EDX mapping, confirmed the successful generation of gold nanoparticles. At maximum laser average power (18 W), nanoparticles with an average of 100 nm in size were observed. The key conclusions are (a) Demonstration of a new approach for E-waste recycling using a 355 nm wavelength picosecond width laser ablation process in water to obtain gold nanoparticles. (b) Successful generation of 100 nm average gold nanoparticles with a 90 wt % purity confirmed by SEM–EDX mapping; and (c) Preliminary cost analysis shows recovery at 0.18 USD/g, vastly below market price of 70–80 USD/g for bulk gold, and 100–150 USD/g for nanoparticles, highlighting the method’s economic and environmental advantages. The applicability of the LIBS was shown for monitoring the ablation process over different numbers of passes, which helped confirm the electronic waste sample. These first experimental demonstrations on recovering gold from electronic waste pave the path to an efficient laser-assisted process offering significant advantages over traditional gold recovery techniques, which often involve hazardous chemicals and complex multistep processes. The laser ablation process presents a rapid, chemical-free, and highly selective approach, reducing both environmental impact and operational costs. The ability to recover gold in nanoparticle form further enhances the economic viability of this technique, given the wide range of applications for gold nanoparticles in various fields. The findings underscore the potential of the laser ablation process as a sustainable solution for e-waste recycling, addressing critical global challenges related to the recovery of valuable materials.

## Data Availability

All the data is available throughout the manuscript.
